# Effect of prehospital notification on acute stroke care: a multicenter study

**DOI:** 10.1186/s13049-016-0251-2

**Published:** 2016-04-27

**Authors:** Ming-Ju Hsieh, Sung-Chun Tang, Wen-Chu Chiang, Li-Kai Tsai, Jiann-Shing Jeng, Matthew Huei-Ming Ma, the Taipei EMS Stroke Collaborative Group

**Affiliations:** Department of Emergency Medicine, National Taiwan University Hospital, No. 7, Chung-Shan South Road, Taipei, 100 Taiwan; Department of Neurology, National Taiwan University Hospital, No. 7, Chung-Shan South Road, Taipei, 100 Taiwan; Institute of Epidemiology and Preventive Medicine, National Taiwan University, Taipei, Taiwan

**Keywords:** Prehospital notification, Stroke, Emergency medical service, Thrombolytic therapy

## Abstract

**Background:**

The sooner thrombolytic therapy is given to acute ischemic stroke patients, the better the outcome. Prehospital notification may shorten the time between hospital arrival and brain computed tomography (door-to-CT) and the door-to-needle (DTN) time. This study investigated the effect of prehospital notification on acute stroke care in an urban city in Taiwan.

**Methods:**

This retrospective observational study utilized a prospectively collected dataset from patients treated at 9 hospitals and the emergency medical service (EMS) system in Taipei City from September 1, 2012 to December 31, 2014. During the study period, prehospital notification was performed by emergency medical technicians if the patient met the following criteria: (1) positive Cincinnati Prehospital Stroke Scale (CPSS), (2) symptom onset within 3 h, and (3) a sugar pinprick test result ≥ 60 mg/dL. The demographics, final diagnoses, and data associated with stroke for all patients in the prenotification group and for patients diagnosed with acute stroke within 3 h of symptoms onset were prospectively recorded in the stroke registry. The primary outcome was door-to-CT time and the secondary outcome was DTN time. The sensitivity and positive predictive value (PPV) of prehospital notifications and the association between the volume of patients receiving thrombolytic therapy at individual hospitals and DTN time were also evaluated.

**Results:**

There were 928 patients who presented ≤ 3 h from stroke onset. Among them, 727 (78.3 %) patients were in the prenotification group; of these, more were male, smokers, and presented with severe symptoms, and fewer had a history of prior stroke or cardiac diseases compared to patients in the non-prenotification group. The median door-to-CT time was significantly shorter in the prenotification group than among the non-prenotification group (13 versus 19 min, *p* < 0.001). Prenotification was associated with shorter DTN time (63 versus 68 min, *p* = 0.138). The sensitivity and PPV of prenotification of stroke were 78.3 % and 78.2 %, respectively. The DTN time demonstrated a significant and highly negative association with the volume of patients receiving thrombolytic therapy (Spearman’s correlation coefficient -0.90, *p* < 0.001).

**Discussion:**

In our study, we found prehospital notification was associated with faster door-to-CT scan and shorter DTN time in patients presenting within 3 hours of symptom onset. Such a close collaboration between hospitals and the EMS system gives citizens an in-time emergency care network. Our study revealed that, like in other countries, prehospital notification for stroke patients improved in-hospital stroke care in Taiwan. Our study showed that the sensitivity and PPV of prenotification decisions according to our CPSS-based criteria was comparable with those in other studies. Our study also found that DTN time was shorter in the hospital that treated a greater volume of patients with thrombolytic therapy. A multicenter collaboration program is needed to help those hospitals with relatively lower stroke patient volume to set up interventions that have been proven to improve stroke care.

**Conclusions:**

Prehospital notification of stroke can significantly shorten door-to-CT time and improve acute stroke care in Taiwan.

## Background

Stroke is the third leading cause of death and major disability in adults worldwide [1–3]. Intravenous tissue plasminogen activator (tPA) has been shown to be beneficial for patients with acute ischemic stroke, but it can only be given within 3–4.5 h of stroke onset [4, 5]. Studies have shown that the sooner that thrombolytic therapy is given to stroke patients, the better the functional outcome and the lower the complication rate [6–8]. Therefore, more efforts are needed to shorten the time between the onset of stroke symptoms and the initiation of thrombolytic therapy. The American Heart Association (AHA) and the American Stroke Association (ASA) guidelines recommend that emergency medical service (EMS) systems should be utilized once stroke is suspected, because EMS personnel can provide optimal prehospital stroke care and can transport patients to stroke centers [9]. Other research has outlined some parameters for measuring the quality of EMS systems and hospitals for stroke care. It was also suggested that these quality parameters ought to be used to continuously monitor prehospital and in-hospital activities [9, 10].

Prehospital notification by EMS personnel can mobilize the resources of the receiving hospital before patient arrival and has been shown to speed up in-hospital management for stroke patients, such as medical assessment, brain imaging, and laboratory studies [11–21]. Nevertheless, there have been no studies that have evaluated the effect of prehospital notification on acute stroke care in Taiwan. In addition, there is some discrepancy in studies describing the association between prehospital notification and door-to-needle (DTN) time [12–14, 16, 17, 19]. Therefore, the aim of our study was to evaluate the effect of prehospital notification on in-hospital timeliness of care for stroke patients in Taipei, a metropolitan city of Taiwan.

## Methods

### Setting

Data was collected from patients treated at nine acute care hospitals in Taipei City with the cooperation of the local EMS system. These participating hospitals receive about 55 % of the 80,000 to 90,000 patients served by the EMS system in Taipei City annually, and these hospitals routinely provide thrombolytic therapy for stroke patients. The Fire Department of Taipei City is responsible for the EMS system. In the catchment area of the participating hospitals, the EMS system is a two-tier system consisting of 41 basic life support units and 4 advanced life support (ALS) units. In these units, there were 591 emergency medical technicians (EMTs) qualified as EMT-Intermediate who had received 280 h of training, and 84 paramedics who had completed 1280 h of training during the study period. No strict rule was implemented to ask the dispatcher to dispatch an ALS unit for patients with a suspected stroke, except when the patients were identified as having a critical condition. Most patients suspected as stroke were served by EMT-Intermediates. A close collaboration between hospitals and the EMS system when providing stroke patient care had not been well established in Taipei until recent years. Since 2011, the EMS system have used new prenotification criteria, which include: (1) a positive finding on the Cincinnati Prehospital Stroke Scale (CPSS); (2) symptom onset within 3 h; and (3) a blood glucose level ≧ 60 mg/dL via a pinprick test. One patient has a positive finding on the CPSS if any one of the following abnormalities is shown: facial palsy, arm weakness, and speech abnormalities. The method to perform the CPSS was the same as those in the original study [22]. These criteria were adopted and modified based on other prehospital stroke scales found in the literature, such as the CPSS, the Los Angeles prehospital stroke screen (LAPSS), the Ontario prehospital stroke screening tool (OPSS), and the Melbourne Ambulance Stroke Screen (MASS) [22–25], and have been validated in a previous study [26]. If EMTs at the scene suspected that a patient was having a stroke, the patient would be examined to see whether he/she met the criteria. If the patient met the new prenotification criteria, the EMT would perform prehospital notification on the way to the receiving hospital. Then, the patient would be sent to the hospital as soon as possible. After receiving the prehospital notification, the personnel at the emergency department (ED) activated standard in-hospital protocols before the patient arrived. All protocols in participating hospitals attempted to establish a fast-track so that a prenotified patient took first priority for medical assessment by emergency physicians, brain imaging, and laboratory studies. However, there were some differences among the protocols; for example, in some hospitals, neurologists were consulted after intracranial hemorrhage was ruled out, but they were consulted before brain image examinations in others.

Before the prenotification criteria were utilized, all of the EMTs participated in a stroke education program, including symptom identification and care skills, in different time frames. During the 2-h education course, EMTs were taught how to perform the CPSS and check blood glucose level by pinprick test on presumed stroke patients. We taught the same method to perform CPSS from the original study [22]. In addition, the skills of querying the time of symptom onset were also taught and practiced in the course.

Although we encouraged EMTs to utilize the prenotification criteria for all patients whose complaints included any of the warning signs of stroke, as recommended by the AHA guidelines [9], it was up to the EMT’s discretion whether the prenotification criteria were used for any particular patient.

### Study population and data collection

The study period was from September 1, 2012 to December 31, 2014. The inclusion criteria for study subjects were as follows: (1) patients over age 20 with discharge diagnoses of stroke or transient ischemic attack (TIA) and arriving at the ED within 3 h of symptom onset (≤3 h) via EMS; (2) patients receiving prenotification without having discharge diagnoses of stroke or TIA ≤ 3 h. Patients without prehospital or in-hospital data were excluded from our study.

The Taipei EMS stroke registry was established to measure, track, and improve stroke care beginning in September 2012. All 9 participating hospitals joined the registry. Trained hospital staff prospectively identified stroke patients over age 20 who arrived at the ED within 3 h of symptom onset via EMS, as well as all patients arriving with prehospital notification. Staff then collected data on demographics and prehospital and in-hospital quality indicators and management using a standardized, web-based data collection tool.

We extracted the registry data during the study period. The extracted data had been processed before we acquired it from the dataset, and thus the identity of each patient could not be identified. From the web-based registry, we collected data regarding age, sex, underlying diseases, scores on the National Institutes of Health Stroke Scale upon ED arrival, prehospital and in-hospital management time, and reasons why thrombolytic therapy was not given.

### Study outcomes and variables

Our primary outcome was door-to-computed tomography (door-to-CT) time. The door-to-CT time was defined as the period from ED arrival to time of CT completion. The time of CT completion was obtained from the film printout or the digital image of the radiology report. The reason why we chose the door-to-CT time as our primary outcome was that there were few differences in management protocols before patients received CT examinations among hospitals; thus, it eased the effect of the different protocols from different hospitals. The secondary outcome was DTN time. The DTN time was defined as the time interval between ED arrival and time that the patient received their first bolus of tPA. The total prehospital management time included the period of time from the receipt of the call by EMS to the patient’s arrival at the hospital. In addition, we also evaluated the positive predictive value (PPV) and sensitivity of the prenotification calls and tried to understand the reasons why thrombolytic therapy was not given to patients presenting ≤ 3 h after stroke onset. The association between the volume of patients receiving thrombolytic therapy and DTN time in individual hospitals was also evaluated. The study received approval from the institutional review board of National Taiwan University Hospital.

### Statistical analysis

The chi-square test, Student’s *t*-test, and Wilcoxon rank-sum test were used to compare the differences between variables for patients with and without prehospital notification. The PPV, sensitivity of prenotification, and 95 % confidence intervals (CIs) were calculated. Spearman's rank correlation coefficient was used to evaluate the association between median DTN time and the volume of patients receiving thrombolytic therapy in individual hospitals. SAS software (Version 9.2, SAS Institute Inc., Cary, NC) was used for statistical analyses. A two-tailed p-value less than 0.05 was defined as statistically significant.

## Results

**Fig. 1:**
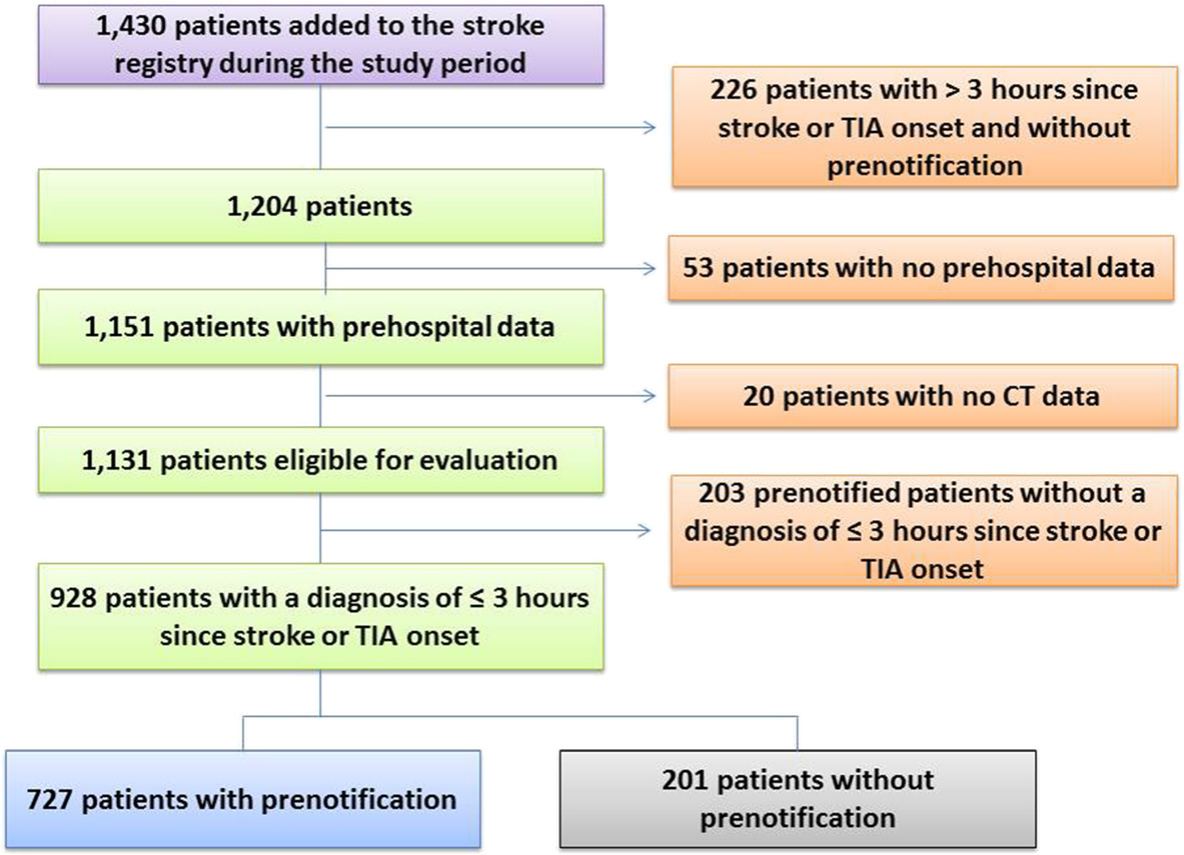
Flowchart of participants included in the study. TIA: transient ischemic stroke

During the study period, there were 1,430 records entered into the registry. There were no patients aged less than 20 years old in the records. We excluded patients who presented > 3 h after stroke or TIA and did not arrive at the hospital with prenotification (*n* = 226), whose records did not include prehospital data (*n* = 53), and whose records did not include documented CT time (*n* = 20). After these exclusions, there were 1,131 patients who were included in the results (Fig. 1). The rate of data missing was 6.1 % (73/1204). Among them, 203 prenotified patients had other discharge diagnoses other than ischemic stroke, hemorrhagic stroke, and TIA within 3 h of symptom onset. Therefore, there were 928 patients presenting ≤ 3 h after symptom onset and diagnosed with stroke or TIA in our study.

Of the 928 patients who presented ≤ 3 h after symptom onset and who were diagnosed with stroke or TIA, 727 patients received prenotification, while the others did not. The characteristics of these patients are shown in Table 1. The prenotification group included more males, more recent smokers, and fewer patients with underlying diseases such as prior ischemic stroke, hypercholesterolemia, and cardiac diseases in comparison with the no-prenotification group. The prenotification group also had a higher stroke severity. In terms of the primary outcome, the prenotification group had a significantly shorter door-to-CT time than the no-prenotification group (13 versus 19 min, *p* < 0.001) (Table 2). In regards to the secondary outcome, the prenotification group was associated with a shorter DTN time (63 versus 68 min, *p* = 0.138) and a higher percentage of DTN time ≤ 60 min (45.1 % versus 28.0 %, *p* = 0.110). Both groups had similar times in regards to total prehospital management time.

**Table 1:** Demographics of patients with and without prenotification

	Prenotification (*n* = 727)	No prenotification (*n* = 201)	*p*-value
Male	469 (64.5 %)	104 (51.7 %)	0.001
Mean age, years	69.0 ± 15.1	70.6 ± 16.2	0.204
Past history			
Prior ischemic stroke	112 (15.4 %)	39 (19.4 %)	0.174
Hypertension	426 (58.6 %)	113 (56.2 %)	0.545
Hypercholesterolemia	72 (9.9 %)	33 (12.4 %)	0.001
Cardiac disease	167 (23.0 %)	69 (34.3 %)	0.001
Chronic renal disease	11 (1.5 %)	6 (3.0 %)	0.229
Diabetes mellitus	140 (19.3 %)	46 (22.9 %)	0.255
Malignancy	35 (4.8 %)	12 (6.0 %)	0.508
Alcohol intake in last 2 years	32 (4.4 %)	8 (4.0 %)	0.795
Smoking in last 2 years	78 (10.7 %)	15 (7.5 %)	0.172
Median NIHSS score	16 (9–22)	12.5 (8–18)	0.081
Stroke type			
Hemorrhagic stroke	250 (34.4 %)	66 (32.8 %)	<0.001
Ischemic stroke	433 (59.6 %)	105 (52.2 %)	
Transient ischemic attack	44 (6.1 %)	30 (14.9 %)	

Values are a number (percentage) or mean ± standard deviation

**Table 2:** Prehospital and in-hospital management time for patients with and without prenotification

	Prenotification (*n* = 727)	No prenotification (*n* = 201)	*p*-value
Total prehospital time, median	22.5 (18.5–26.0)	22 (17.5–26.5)	0.433
Door to CT time, median	13 (10.0–18.0)	19 (13.0–34.0)	<0.001
Door to CT time ≤ 25 min	660 (90.8 %)	125 (62.2 %)	<0.001
Door to needle time, median	63 (49.0–79.0)	68 (54.0–86.0)	0.138
Door to needle time ≤ 60 min	65 (45.1 %)	7 (28.0 %)	0.110
Administering thrombolytic therapy	144 (19.8 %)	25 (12.4 %)	0.017

Values are a number (percentage) or median (upper quartile, lower quartile)

**Fig. 2:**
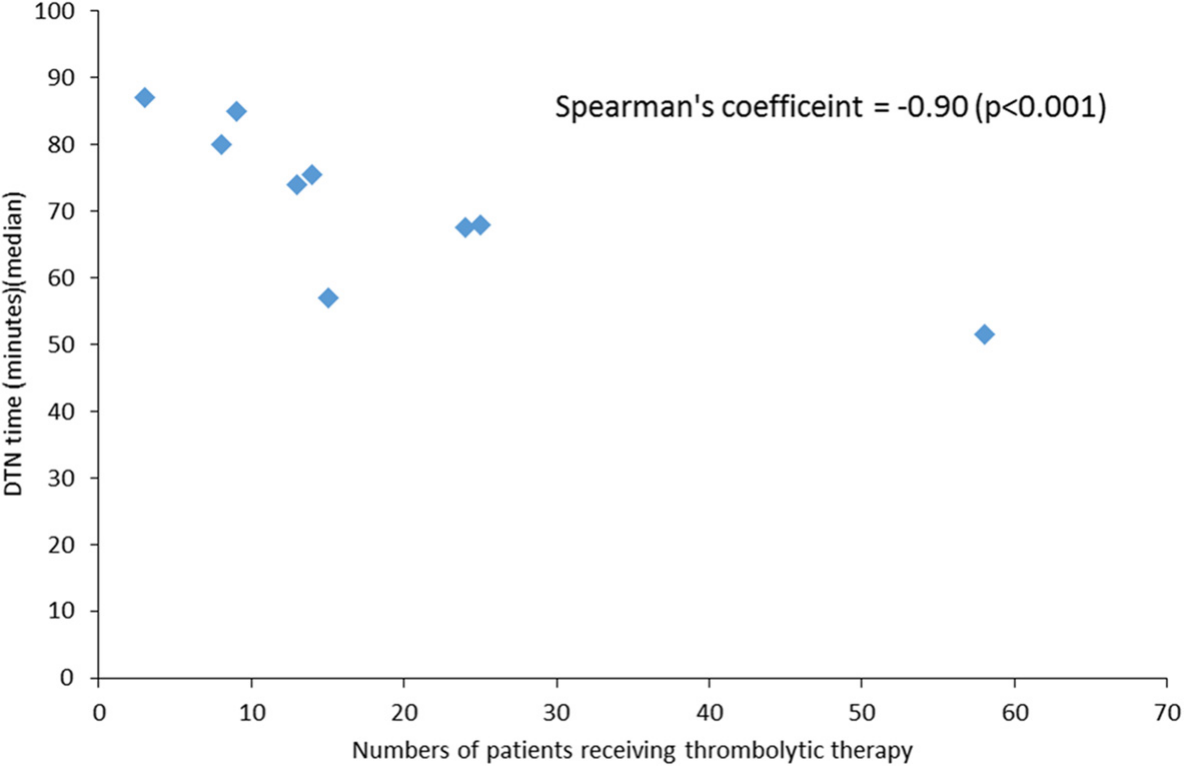
The relationship between the number of patients receiving thrombolytic therapy and the median door-to-needle (DTN) time in the hospitals joining the study. Each data point referred to each hospital site. The median DTN time had a highly negative association with the volume of patients receiving thrombolytic therapy

The PPV and sensitivity of the prehospital notification criteria were 78.2 % (727/930, 95 % CI: 75.4–80.8) and 78.3 % (727/928, 95 % CI: 75.5–80.9). Among 203 prenotified patients without diagnoses of stroke or TIA ≤ 3 h after onset, the most common discharge diagnoses were stroke or TIA > 3 h after onset (57.1 %) and seizure (19.2 %) (Table 3). Among the 685 patients who presented ≤ 3 h after stroke onset and did not receive thrombolytic therapy, the most frequent three reasons were: hemorrhagic stroke or hemorrhagic transformation (46.1 %), age greater than 80 years old (22.9 %), and prompt symptom recovery or minor stroke (13.0 %) (Table 4). Ten (1.5 %) patients who were suitable for thrombolytic therapy refused the treatment. The DTN times among participating hospitals were also different, ranging from 51.5 min to 87 min, and the DTN time was significantly and highly negatively associated with the volume of patients receiving thrombolytic therapy (Spearman’s correlation coefficient -0.90, *p* < 0.001) (Fig. 2).

**Table 3:** Other diagnoses (excluding ≤ 3 h acute cerebrovascular disease) among patients with prenotification (*n* = 203)

Diagnoses	n (%)
>3 hours since stroke onset or TIA^a^	116 (57.1 %)
Seizure	39 (19.2 %)
Hypoglycemia	4 (2.0 %)
Peripheral vertigo or dizziness	2 (1.0 %)
Prior stroke	5 (2.5 %)
Traumatic brain injury	3 (1.5 %)
Syncope	5 (2.5 %)
Intracranial tumor	5 (2.5 %)
Drug or alcohol overdose	3 (1.5 %)
Hepatic encephalopathy	4 (2.0 %)
Serotonin syndrome	1 (0.5 %)
Hydrocephalus	1 (0.5 %)
Aortic dissection	2 (1.0 %)
Weakness due to other medical problems	13 (6.4 %)

^a^TIA: transient ischemic attack

**Table 4:** Reasons thrombolytic therapy was not administered to patients presenting within 3 h of stroke onset

Reasons	Prenotification (*n* = 539)	No prenotification (*n* = 146)	Total (*n* = 685)
Hemorrhagic stroke or hemorrhagic transformation	250 (46.4 %)	66 (45.2 %)	316 (46.1 %)
Older than 80 years of age	117 (21.7 %)	40 (27.4 %)	157 (22.9 %)
Rapid recovery or minor symptoms	70 (13.0 %)	19 (13.0 %)	89 (13.0 %)
Very severe symptoms/signs	28 (5.2 %)	9 (6.2 %)	37 (5.4 %)
Stroke or serious head injury within 3 months	11 (2.0 %)	2 (1.4 %)	13 (1.9 %)
Prior stroke accompanied with diabetes mellitus history	7 (1.3 %)	3 (2.1 %)	10 (1.5 %)
Seizure during stroke onset	8 (1.5 %)	2 (1.4 %)	10 (1.5 %)
Patient or family refused therapy	9 (1.7 %)	1 (0.7 %)	10 (1.5 %)
Uncontrolled hypertension	8 (1.5 %)	0 (0 %)	8 (1.2 %)
International normalized ratio >1.7	4 (0.7 %)	3 (2.1 %)	7 (1.0 %)
Known vascular malformation, aneurysm, or brain tumor	7 (1.3 %)	0 (0 %)	7 (1.0 %)
More than 4.5 hours before thrombolytic therapy given	6 (1.1 %)	0 (0 %)	6 (0.9 %)
Other	14 (2.6 %)	1 (0.7 %)	15 (2.2 %)

## Discussion

In our study, we found prehospital notification was associated with faster door-to-CT scan in patients presenting within 3 h of symptom onset. It saved time from arrival to CT completion and gave both doctors and patients more time to make further evaluations and decisions for a disease with time-limited treatment options. Such a close collaboration between hospitals and EMS systems gives citizens an in-time emergency care network. Our study revealed that, like in other countries [11–18, 21], prenotification for stroke patients improved in-hospital stroke care in Taiwan.

Prenotification was also associated with shorter DTN time in our study. In the literature, prenotification shortened DTN time significantly in some studies [13, 14, 16, 17], but it did not show the same effect in other studies [12, 19]. Such discrepancies regarding the effect of prenotification on DTN time among these studies may be caused by small sample sizes or may be related to the fact that the time that thrombolytic therapy is begun is affected not only by the speed of laboratory studies, imaging studies, and medical evaluation by medical personnel, but also by communication between doctors and patients or family members, and the speed that decisions are reached by doctors, patients, and family members. Poor communication or hesitation to make a decision could delay the initiation of treatment. These unmeasured confounders suggest that the association between prenotification and DTN time is inconclusive. Our results suggest that to further shorten the DTN time in our community, more efforts are needed to improve the process during the period between CT completion and thrombolysis, because more time, which had the potential to be saved, was consumed during this period.

Our study demonstrated that the PPV and sensitivity of prenotification decisions were high, both reaching 80 %. In the literature, the PPV of the CPSS ranged from 40 % to 85 %, and the sensitivity ranged between 44 %–95 % [22, 25, 27–32]. The PPV and sensitivity of prenotification decisions according to our CPSS-based criteria was comparable with those in other studies. Although our study showed that prenotification decisions had high PPV and sensitivity, to further improve the accuracy of prenotification decisions, more efforts are needed to develop periodic refresher programs to maintain important skills regarding clarification of the onset time of symptoms, because the most frequent diagnosis for patients with an inaccurate prenotification decision was stroke occurring more than 3 h earlier. In addition, the final diagnosis for about 20 % of patients with an incorrect prenotification decision was seizure. In the previous validated prehospital stroke screening tools, such as LAPSS, OPSS, MASS and the Recognition of Stroke in the Emergency Room scale, history of seizure or seizure at onset was one of the criteria [23–25, 33]. In order to further improve the PPV of prenotification decisions, it may be useful to add a seizure criterion in order to eliminate patients with seizure at onset and to improve the skills of EMTs to identify seizures.

Our study showed that very few patients eligible for thrombolytic therapy refused the therapy. This suggested that most patients accepted thrombolytic therapy after physicians explained the benefits of the treatment in spite of the risk of intracerebral hemorrhage. This result hints that emergency care staff must pay attention to identify stroke patients and to give appropriate therapy. In addition, the stroke patients included in our study had moderate to severe symptoms, and it was speculated that stroke patients who suffered from minor symptoms might arrive at hospitals by themselves instead of EMS. To increase EMS utilization by stroke patients, more efforts are needed not only to enable the citizens to identify stroke symptoms, but also to emphasize the importance of calling ambulance to the public.

Our study also showed that DTN time was shorter in the hospital that treated a greater volume of patients with thrombolytic therapy. One multicenter study found that the annual volume of patients receiving thrombolysis affected the DTN time much more than the years of treatment [34]. Another study also found that higher annual volume was associated with lower stroke mortality [35]. Therefore, a multicenter collaboration program is needed to help those hospitals with relatively lower stroke patient volume to set up interventions that have been proven to improve stroke care.

There were some limitations to our study. First, our study was performed in a metropolitan city, and the study results might not be generalizable to rural areas. Second, all of the participating hospitals had standard in-hospital protocols for treating stroke patients. The effect of prenotification on stroke care, as demonstrated in our study, might be different in hospitals without such protocols. Nevertheless, our study revealed that prehospital notification was a useful strategy when a close collaboration was developed between well-trained and organized EMS systems and hospitals.

## Conclusions

Prehospital notification shortened door-to-CT time significantly, and was also associated with improved DTN time. EMTs also exhibited good PPV and sensitivity when applying prenotification criteria but also had some room for improvement. Our study showed that prehospital notification is a feasible strategy for better stroke care in Taiwan.

## Abbreviations

tPA: tissue plasminogen activator
AHA: American Heart Association
ASA: American Stroke Association
EMS: emergency medical service
DTN: door-to-needle
CPSS: Cincinnati Prehospital Stroke Scale
LAPSS: Los Angeles prehospital stroke screen
OPSS: Ontario prehospital stroke screening tool
MASS: Melbourne Ambulance Stroke Screen
EMTs: emergency medical technicians
ED: emergency department
PPV: positive predictive value
CI: confidence interval

## References

[1] Sarti C, Rastenyte D, Cepaitis Z, Tuomilehto J (2000). International trends in mortality from stroke, 1968 to 1994. Stroke..

[2] Foulkes MA, Wolf PA, Price TR, Mohr JP, Hier DB (1988). The stroke data bank: design, methods, and control characteristics. Stroke..

[3] Jeng JS, Su TC (2007). Epidemiological studies of cerebrovascular diseases and carotid atherosclerosis in Taiwan. Acta Neurol Taiwan..

[4] Tissue plasminogen activator for acute ischemic stroke (1995). The National Institute of Neurological Disorders and Stroke rt-PA Stroke Study Group. N Engl J Med.

[5] Hacke W, Kaste M, Bluhmki E, Brozman M, Dávalos A, Guidetti D (2008). Thrombolysis with alteplase 3 to 4.5 hours after acute ischemic stroke. N Engl J Med.

[6] Hacke W, Donnan G, Fieschi C, Kaste M, von Kummer R, Broderick JP (2004). Association of outcome with early stroke treatment: pooled analysis of ATLANTIS, ECASS, and NINDS rt-PA stroke trials. Lancet..

[7] Lees KR, Bluhmki E, von Kummer R, Brott TG, Toni D, Grotta JC (2010). Time to treatment with intravenous alteplase and outcome in stroke: an updated pooled analysis of ECASS, ATLANTIS, NINDS, and EPITHET trials. Lancet..

[8] Fonarow GC, Smith EE, Saver JL, Reeves MJ, Bhatt DL, Grau-Sepulveda MV (2011). Timeliness of tissue-type plasminogen activator therapy in acute ischemic stroke: patient characteristics, hospital factors, and outcomes associated with door-to-needle times within 60 minutes. Circulation..

[9] Jauch EC, Saver JL, Adams HP, Bruno A, Connors JJ, Demaerschalk BM (2013). Guidelines for the early management of patients with acute ischemic stroke: a guideline for healthcare professionals from the American Heart Association/American Stroke Association. Stroke..

[10] Acker JE, Pancioli AM, Crocco TJ, Eckstein MK, Jauch EC, Larrabee H (2007). Implementation strategies for emergency medical services within stroke systems of care: a policy statement from the American Heart Association/American Stroke Association Expert Panel on Emergency Medical Services Systems and the Stroke Council. Stroke..

[11] Sheppard JP, Mellor RM, Greenfield S, Mant J, Quinn T, Sandler D (2015). The association between prehospital care and in-hospital treatment decisions in acute stroke: a cohort study. Emerg Med J..

[12] McKinney JS, Mylavarapu K, Lane J, Roberts V, Ohman-Strickland P, Merlin MA (2013). Hospital prenotification of stroke patients by emergency medical services improves stroke time targets. J Stroke Cerebrovasc Dis..

[13] Casolla B, Bodenant M, Girot M, Cordonnier C, Pruvo JP, Wiel E (2013). Intra-hospital delays in stroke patients treated with rt-PA: impact of preadmission notification. J Neurol..

[14] Lin CB, Peterson ED, Smith EE, Saver JL, Liang L, Xian Y (2012). Emergency medical service hospital prenotification is associated with improved evaluation and treatment of acute ischemic stroke. Circ Cardiovasc Qual Outcomes..

[15] Patel MD, Rose KM, O’Brien EC, Rosamond WD (2011). Prehospital notification by emergency medical services reduces delays in stroke evaluation. Findings from the North Carolina stroke care collaborative. Stroke.

[16] Bae HJ, Kim DH, Yoo NT, Choi JH, Huh JT, Cha JK (2010). Prehospital notification from the emergency medical service reduces the transfer and intra-hospital processing times for acute stroke patients. J Clin Neurol..

[17] Kim SK, Lee SY, Bae HJ, Lee YS, Kim SY, Kang MJ (2009). Pre-hospital notification reduced the door-to-needle time for iv t-PA in acute ischaemic stroke. Eur J Neurol..

[18] Abdullah AR, Smith EE, Biddinger PD, Kalenderian D, Schwamm LH (2008). Advance hospital notification by EMS in acute stroke is associated with shorter door-to-computed tomography time and increased likelihood of administration of tissue-plasminogen activator. Prehosp Emerg Care..

[19] Quain DA, Parsons MW, Loudfoot AR, Spratt NJ, Evans MK, Russell ML (2008). Improving access to acute stroke therapies: a controlled trial of organised pre-hospital and emergency care. Med J Aust..

[20] Mosley I, Nicol M, Donnan G, Patrick I, Kerr F, Dewey H (2007). The impact of ambulance practice on acute stroke care. Stroke.

[21] Bray JE, Martin J, Cooper G, Barger B, Bernard S, Bladin C (2005). An interventional study to improve paramedic diagnosis of stroke. Prehosp Emerg Care..

[22] Kothari RU, Pancioli A, Liu T, Brott T, Broderick J (1999). Cincinnati Prehospital Stroke Scale: reproducibility and validity. Ann Emerg Med..

[23] Kidwell CS, Starkman S, Eckstein M, Weems K, Saver JL (2000). Identifying stroke in the field. Prospective validation of the Los Angeles prehospital stroke screen (LAPSS). Stroke.

[24] Chenkin J, Gladstone DJ, Verbeek PR, Lindsay P, Fang J, Black SE (2009). Predictive value of the Ontario prehospital stroke screening tool for the identification of patients with acute stroke. Prehosp Emerg Care..

[25] Bray JE, Martin J, Cooper G, Barger B, Bernard S, Bladin C (2005). Paramedic identification of stroke: community validation of the Melbourne Ambulance Stroke Screen. Cerebrovasc Dis..

[26] Hsieh MJ, Tang SC, Ko PC, Chiang WC, Tsai LK, Chang AM, et al. Improved performance of new prenotification criteria for acute stroke patients. J Formos Med Assoc. 2015; doi:10.1016/j.jfma.2015.03.007 10.1016/j.jfma.2015.03.007 25886861

[27] Ramanujam P, Guluma KZ, Castillo EM, Chacon M, Jensen MB, Patel E (2008). Accuracy of stroke recognition by emergency medical dispatchers and paramedics - San Diego experience. Prehosp Emerg Care..

[28] Frendl DM, Strauss DG, Underhill BK, Goldstein LB (2009). Lack of impact of paramedic training and use of the Cincinnati Prehospital Stroke Scale on stroke patient identification and on-scene time. Stroke..

[29] Bray JE, Coughlan K, Barger B, Bladin C (2010). Paramedic diagnosis of stroke: examining long-term use of the Melbourne Ambulance Stroke Screen (MASS) in the field. Stroke..

[30] Studnek JR, Asimos A, Dodds J, Swanson D (2013). Assessing the validity of the Cincinnati Prehospital Stroke Scale and the medic prehospital assessment for code stroke in an urban emergency medical services agency. Prehosp Emerg Care..

[31] Bergs J, Sabbe M, Moons P (2010). Prehospital stroke scales in a Belgian prehospital setting: a pilot study. Eur J Emerg Med..

[32] Asimos AW, Ward S, Brice JH, Rosamond WD, Goldstein LB, Studnek J (2014). Out-of-hospital stroke screen accuracy in a state with an emergency medical services protocol for routing patients to acute stroke centers. Ann Emerg Med..

[33] Nor AM, Davis J, Sen B, Shipsey D, Louw SJ, Dyker AG (2005). The Recognition of Stroke in the Emergency Room (ROSIER) scale: development and validation of a stroke recognition instrument. Lancet Neurol..

[34] Strbian D, Ahmed N, Wahlgren N, Lees KR, Toni D, Roffe C (2015). Trends in door-to-thrombolysis time in the safe implementation of stroke thrombolysis registry: effect of center volume and duration of registry membership. Stroke..

[35] Saposnik G, Baibergenova A, O'Donnell M, Hill MD, Kapral MK, Hachinski V (2007). Hospital volume and stroke outcome: does it matter?. Neurology..

